# Supplementary material for: Non-Secretor Status Due to FUT2 Stop Mutation Is Associated with Reduced Rotavirus Infections but Not with Other Enteric Pathogens in Rwandan Children
Source: Microorganisms. 2025 May 3;13(5):1071. doi: 10.3390/microorganisms13051071 (PMC12114204; doi:10.3390/microorganisms13051071)
Supplement: Supplementary file 1 [file microorganisms-13-01071-s001.zip › microorganisms-3568208-supplementary.pdf]

Supplementary Table S1. Primers and probes for real-time PCR

	Forward primer	Reverse primer	Probe
Norovirus GII	TGGAYTTTTAYGTGCCCAG	CGACGCCATCTTCATTCAC	VIC-AGCCAGATTGCGATCGCCC
Rotavirus	AACCATCTACACATGACCCTCTATGA	GGTCACATAACGCCCCTATAGC	FAM-CAATAGTTAAAAGCTAACACTGTCAAA
	AACCATCTTCACGTAACCCTCTATGA		
Astrovirus	GACTGCWAAGCAGCTTCGTGA	GCTAGCCATCACACTTCTTTGGTCCT	VIC-TCACAGAAGAGCAACTCCATCGCATTTG
Sapovirus	TTGGCCCTCGCCACCTAC	CCCTCCATYTCAAACACTA	VIC-CCRCCTATRAACCA-MGB
	GAYCASGCTCTCGCYACCTAC		
Norovirus GI	TGGCAGGCCATGTTCCGCT	TTTGKTGGGGCGTCCTTAGAC	VIC-ATTGCGATCTCCTGTCCA-MGB
		CGCTTGATGTAGCGTCCTTAGAC	
Campylobacter	ATGCAAACCATAATTGGGTTTCAAC	CGAGTATCAGCAACTTCTTCTACAGCT	NED-TTGCCACCAAAACCAAAACTMGB
Salmonella	CGGGTTGCGTTATAGGTCTGA	TGAAATACGATGCGAACAACATC	VIC-AATACTGCGCTGCCAGAT-MGB
ETEC-*estA*	AAGCATGAATAGTAGCAATTACTGCT	TTAATAGCACCCGGTACAAGCA	NED-AACAACACAATTCAC-MGB
ETEC-*eltB*	TCCGGCAGAGGATGGTTACA	CCAGGGTTCTTCTCTCCAAGC	FAM-AGCAGGTTTCCCACCGGATCACC
Shigella/EIEC	ACCGGCGCTCTGCTCTC	GCAATGTCCTCCAGAATTTCG	JOE-CTGGGCAGGGAAATGTTCCGCC
Cryptosporidium parvum/hominis	CAAATTGATACCGTTTGTCCTTCTG	TGGTGCCATACATTGTTGTCCT	NED-TGTCCTCCTGGATTCA-MGB
EPEC-*eae*	ACATGACCGATGACAAGGCA	CGCGACTGAAGCTGGCTAC	JOE-TCGCCGCCTGTTGTGCCG
EPEC-*bfpA*	GGTCTGTCTTTGATTGAATCTGCA	GCAGACTGGTAGTAAAACATCACACC	FAM-GCGCTTGCTGCCACCGTTACCG
Adenovirus 40/41	TGCCCGCGCCACCGAT	GAGCCACAGTGGGGTTTCTG	NED-CCAGGCTGAAGTACG

For some targets a mixture of two forward or reverse primers were used to mach sequence variability in published sequences.
MGB, minor groove binding quencher. BHQ1 (black hole quencher 1) was used in non-MGB-probes. All probe concentrations were 200 nM. Primer concentrations were 900 nM for astrovirus and norovirus GI, 300 nM for all other agents
